# Influence of Water Intake and Balance on Body Composition in Healthy Young Adults from Spain

**DOI:** 10.3390/nu11081923

**Published:** 2019-08-15

**Authors:** Ana Isabel Laja García, Carmen Moráis-Moreno, Mª de Lourdes Samaniego-Vaesken, Ana M. Puga, Teresa Partearroyo, Gregorio Varela-Moreiras

**Affiliations:** Departamento de Ciencias Farmacéuticas y de la Salud, Facultad de Farmacia, Universidad San Pablo-CEU, CEU Universities, Urbanización Montepríncipe, 28925 Alcorcón, Madrid, Spain

**Keywords:** water intake, water balance, weight management, obesity, overweight, body composition

## Abstract

The increasing prevalence of overweight and obesity has become an epidemic public health problem worldwide. In the last years, several investigations have suggested that water intake and retention could have important implications for both weight management and body composition. However, there is a lack of information about this issue globally, and mainly specifically in Spain. Thus, the aim of this study was to analyze the association between hydration status and body composition in a sample of healthy Spanish adults. The study involved 358 subjects, aged 18–39 years. The recently validated “hydration status questionnaire” was used to assess their water intake, elimination, and balance. Anthropometric measurements were performed according to the recommendations of the International Standards for Anthropometric Assessment (ISAK). Body composition variables were acquired by bioelectrical impedance analysis. Differences in anthropometric and body composition variables were assessed through the ANOVA test and considered significant at *p* < 0.05. Fluid intake was correlated with body water content. Inverse associations between water consumption, normalized by weight, with body weight, body fat mass, and waist circumference were found. Moreover, according to water balance, significant differences in body water content in females were observed. In conclusion, higher fluid intake seems to be related with a healthier body composition. Therefore, the improvement of water intake and water balance could be useful for overweight and obesity prevention, although further studies are needed to confirm the present findings.

## 1. Introduction

It is well known that the rising prevalence of overweight and obesity has become a public epidemic around the world [1]. Particularly, in Spain, in the last thirty years, the prevalence of overweight and obesity has increased from 25.6% and 7.9% in women and 38.4% and 6.9% in men, to 30.1%/16.7% and 44.3%/18.2%, respectively [2]. Overweight and obesity has a multifactorial etiology involving genetic predisposition, and environmental and behavioral factors [3]. In addition, obesity is associated with and contributes to a shortened life span, type 2 diabetes mellitus, cardiovascular disease, some cancers, kidney disease, obstructive sleep apnea, gout, osteoarthritis, and hepatobiliary disease, among others [3]. Therefore, the first step to develop prevention is to further understand the obesity–environmental interrelationships. The fact that their classical determinants (diet and physical activity) do not sufficiently explain this situation make it necessary to examine other possible factors which could be implicated [4,5]. In this regard, several investigations have suggested that water intake (WI) and water balance (WB) could have important implications for both weight management and body composition [6,7,8,9].

In recent years, the role of an adequate hydration status (HS) in human health has acquired great interest in research [10,11,12]. Nowadays, water, which is the most abundant component of the human body, comprising approximately 45%–75% of body weight depending on age and physiological status [13,14], is considered an essential nutrient [14]. Some of its functions are transporting nutrients, regulating body temperature, being solvent for many organic and inorganic materials, lubricating joints and internal organs, and providing structure to cells and tissues, among others. Therefore, water is of such importance that humans could only survive 2 to 4 days without it [13]. Despite the key role that an adequate HS exerts in health and wellness, available data of water consumption amongst the population shows a high prevalence of inadequate hydration habits [15]. Particularly, data from the Spanish ANIBESstudy (“anthropometric data, macronutrients and micronutrients intake, practice of physical activity, socioeconomic data and lifestyles in Spain”) [16] showed that more than 75% of participants did not achieve the European Food Safety Association (EFSA) WI recommendations (2.5 L/day for men and 2.0 L/day for woman). 

Drinking high amounts of water is commonly and popularly related to weight loss; however, until recent years, limited scientific evidence was available to justify this relationship. In this regard, recently, several investigations [17,18,19] have tried to support this association by concluding that there is a positive relation between WI, weight management, and body composition, although the responsible mechanisms remain unclear. It has been suggested that drinking water increases the rate of lipolysis and energy expenditure by means of sympathetic stimulus and thermogenesis induction [20,21,22]. Furthermore, short-term effects of water consumption include increased satiety and, consequently, reduced hunger feeling [23]. In this context, some investigations have demonstrated an increase of energy intake when pre-meal water ingestion was removed [17,24,25]. Finally, drinking water and an adequate HS were also related to healthier dietary patterns [26]. Noteworthy, the consumption of foods with high water content, such as fruits and vegetables, can contribute to increased daily WI and, as a consequence, improve HS [27].

The evidence of the important role that HS and WI plays on body weight and body composition make it necessary to develop new studies which confirm and aid the deepening of the knowledge of this relation, with the purpose of establishing public health measures which may contribute to reduce current rates of overweight and obesity. In this regard, it is important to mention that nowadays there is lack of consensus about the best method to estimate WI [28] and the accurate estimation of HS implies a great difficulty [29,30]. In fact, there is no “gold standard”, and the techniques and methods available are expensive, invasive and/or complicated, making it difficult to apply at population level [29,30,31,32,33]. Recently, our research group designed a questionnaire entitled “The hydration status questionnaire” (HSQ) [34], which has been demonstrated to be valid for the evaluation of WI and HS of a healthy adult population, as well as an adaptation for an adolescent-young population [35]. 

For all the aforementioned, the hypothesis of this study was that an adequate WI and HS could play a beneficial role in weight and body composition. Thus, the aim of the present study was to examine, for the first time, the effect of WI and WB, estimated by means of the previously mentioned HSQ, on body composition in healthy young adults from Spain. 

## 2. Materials and Methods

This cross-sectional study started in October 2015 and was developed during two consecutive years, finishing in October 2017. Volunteers were recruited from Montepríncipe Campus of CEU San Pablo University (Madrid, Spain). The inclusion criteria were: Individuals who were (a) mentally and physically healthy and (b) aged 18–39 years. Exclusion criteria were: (a) Suffering from diseases related to HS, including renal impairment, urinary tract infection, WB disease, and diabetes; and/or (b) females who were menstruating during the study. Volunteer recruitment was performed through informative talks, posters, and through the website of the University. 

Ethical approval was granted by the Clinical Research Ethics Committee of the CEU San Pablo University (Madrid). The corresponding ethical code was 106-15. Moreover, the study was performed in accordance with the ethical standards laid down in the 1964 Declaration of Helsinki and its later amendments. Participants were informed about the objectives of the study and the procedures involved, and signed an informed consent prior to their inclusion in the study. All personal data were confidential and only investigators assigned to the project had access to them. In any case, it complied with the General Data Protection Regulation (2016/679).

### 2.1. Study Protocol 

The study included the HS and WI assessment and the anthropometric evaluation of the subjects. Participants completed the HSQ [34], which was previously validated in a healthy adult population and allowed the estimation of WI from food and beverages and water elimination (WE) from urine and feces. Moreover, volunteers also completed the short form of the international physical activity questionnaire (IPAQ-SF) [36] to assess their physical activity and to estimate WE by sweat. WB was calculated as the difference between total WI and total WE. 

The anthropometric evaluation comprised the measurement of weight, by a digital scale with an accuracy of 200 g (SECATM 877); height, measured to the nearest 0.1 cm using a wall-mounted stadiometer (SECA^TM^); and waist circumference (WC), which was measured with a flexible tape (Cescorf™). Anthropometric measurements were performed according to the recommendations of the International Standards for Anthropometric Assessment (ISAK) [37] by level I and II accredited anthropometrists. Weight and height data were used to calculate the body mass index (BMI), or Quetelet index, according to the following formula [38] (weight (Kg)/height^2^ (m)) and classified as underweight, normal weight, overweight, and obese according to the World Health Organization criteria [39]. Lastly, body composition (total body water (TBW), fat body mass (FBM), lean body mass (LBM), and dry lean body mass (DLBM)) were estimated by bioelectrical impedance (BIA) with a Bioscan Spectrum Multifrequency^TM^. All measurements were achieved under fasting conditions, in the same laboratory of the University and under mild temperature conditions (22 ± 2 °C and relative humidity of 50% ± 10%). 

### 2.2. Statistical Analysis

Results are presented as mean and 95% confidence interval. Differences between variables were assessed with the Student’s paired t-test and considered significant at *p* ≤ 0.05. The relation between HS, WI, and anthropometric characteristics was evaluated using Pearson’s (*r*) correlation coefficient. Differences in water consumption and HS according to body BMI were analyzed through ANOVA test and considered significant at *p* ≤ 0.05. Finally, differences in anthropometric and body composition variables, according to WB percentiles (p) (<p25 = 161.7 mL, p25–p50 = 161.7–859.8 mL, p50–p75 = 859.9–1484.5 mL, >p75 = 1484.6 mL) and according to WI normalized by body weight percentiles (<p25 = 39.7 mL/Kg, p25–p50 = 39.8–49.0 mL/Kg, p50–p75 = 49.1–60.1 mL/Kg, >p75 = 60.2 mL/Kg), were also analyzed through ANOVA test and were considered significant at *p* ≤ 0.05. All statistical analyses were performed using SPSS 24.0 Software (IBM Corp., Armonk, NY, USA). 

## 3. Results

### 3.1. Sample Charasteristics

A total of 358 healthy volunteers, 121 males (33.8%) with a mean age of 21.6 (21.0–22.2) years and 237 females (66.2%) with a mean age of 21.6 (21.2–22.1) years, participated in the study. Their anthropometric characteristics are presented in Table 1, where it can be observed that there were significant differences between genders in all the collected variables. 

In the study population, overweight and obesity prevalence was 12.2% and data analyzed by gender showed that it was significantly higher in males when compared to females (23.1% and 6.8%, respectively) (*p* = 0.000). Nevertheless, the prevalence of underweight for the total sample was 7.9%, being higher in females (10.1%) than in males (3.3%). Finally, 79.9% of the sample presented as normal weight (males: 73.6%; females: 83.1%). 

Results of the HSQ sorted by gender are presented in Table 2. It can be observed that WI, drinking water, water from beverages, and total WI were significantly higher in males compared to females. Nevertheless, water from food and WB were significantly higher in females than in males. In addition, significant differences in the amount of WI per body weight from all sources were observed, being higher in females than in males.

### 3.2. Hydration Status and the Correlation with Anthropometric Variables

Drinking water and WI were correlated with TBW in females (*r* = 0.196, *p* = 0.002; *r* = 0.180, *p* = 0.006) and in males (*r* = 0.187, *p* = 0.040; *r* = 0.270, *p* = 0.003), confirming the relationship between HS and body composition. However, no correlations were obtained for the rest of the anthropometric variables. Nevertheless, after data normalization by body weight, associations between WI variables from all sources with all the anthropometrics parameters were found. Associations between WI from different sources and total WI normalized by body weight, with the anthropometric and body composition variables in males and females are presented in Table 3 and Table 4, respectively. As it can be observed, WI normalized by body weight was inversely correlated with weight, BMI, FBM, and WC and, in contrast, positively correlated with TBW percentage. 

In males, inverse associations between WI normalized by body weight with weight, FBM (Kg), and WC and a positive association between this variable and TBW (%) were found. Moreover, water from food normalized by weight was also correlated with weight, FBM (Kg) and with TBW (%).

Differences in WI variables and WB related to BMI are presented in Table 5. Our results showed that overweight/obese males had higher water intakes from beverages than those who were underweight. Nevertheless, in females the volume of WI normalized by body weight as well as water from food normalized by body weight was higher in normal weight and underweight woman than in overweight and obese individuals. Lastly, females who were underweight also had a higher WI normalized by body weight than overweight/obese individuals. 

On the other hand, differences in anthropometric and body composition variables related to WB percentiles were also analyzed and significant differences in TBW of females were observed (p25–p50 = 28.1 (27.6–28.6) L; p75 = 29.2 (28.6–29.9) L, (*p* = 0.04). 

Finally, differences in body composition and anthropometric variables related to WI normalized by body weight were analyzed. Results obtained in females are presented in Table 6, where it can be observed that women who had higher WI normalized by body weight presented a lower weight, BMI, FBM, and WC, whereas, in contrast, their TBW was higher. No significant differences were found in males. 

## 4. Discussion

Results of the present study confirmed the existence of a relation between WI, either from water, other beverages, food, or total WI, with weight and body composition. As expected, a positive correlation between water consumption and TBW was observed. The fact that the other anthropometric variables analyzed (weight, BMI, FBM, and WC) were correlated with total WI and WI from the different sources normalized by body size, but not with their absolute values, brings to light the important role of body size in water requirements. Additionally, other factors such as gender, physical activity, drug consumption [40], energy intake, environmental temperature and humidity, among others [41,42,43], can affect water needs. Therefore, WI recommendations should be as personalized as possible. In this regard, it is important to mention that WI recommendation per body weight has already been established for free-living adults, which suggest a minimum daily intake of 30–45 mL/Kg [44,45]. Around 35.5% of the population included in the current study did not accomplish the highest recommendation, while 8.9% did not even achieve the lowest. Nevertheless, the most widely accepted WI recommendations are the ones established by the EFSA and the Institute of Medicine of the United States of America (IOM) [46,47]. In the current study, both males and females fulfilled the EFSA recommendations (men: 2.5 L/day, women: 2.0 L/day), but only females achieved the ones from IOM (men: 3.7 L/day, women: 2.7 L/day). The reason for the considerable differences between the EFSA and the IOM recommendations remains unclear, considering that both include water from food and water from beverages. 

In recent years, several investigations have shown a positive effect of increasing water consumption in body weight loss and decrease of WC and FBM (%) [7,9,17,18,19,23,24,48]. Moreover, another study observed that women and obese people display indicators of cellular dehydration and have a higher risk of dehydration [49]. Therefore, current available literature indicates that, on the one hand, water consumption can support weight loss and the achievement of an adequate body composition, and on the other, that overweight and obese people tend to be more dehydrated and thus they constitute an at-risk population group. In the current study, inverse associations were observed between WI from all the sources (drinking water, water from beverages, water from food and total WI) normalized by body weight with weight status, FBM, and WC, as well as a positive correlation between WI parameters with TBW in both males and females. When population was categorized by BMI, it was observed that normal weight and underweight females presented higher values of total WI and water from food normalized by body weight than overweight/obese females. Nevertheless, this was not observed in males, given that overweight/obese males drank more water from beverages than underweight ones. Previous investigations have also provided similar results showing that plain water consumption was higher in adults of higher BMI, but it is important to consider that these results were from limited data [50]. Moreover, for a proper interpretation of the results obtained in males, it must be considered that the group of beverages of the HSQ is very wide including plain water, juices, sweet beverages, light beverages, milk and dairy products, infusions, and alcoholic beverages, among others. 

The studied population was also categorized by percentiles of WI normalized by body weight, and it was observed that females who had higher water intakes presented lower weight, BMI, FBM, and WC and, in contrast, greater TBW. The influence of WB in weight and body composition is also of interest, given that it largely determines the HS, which is essential for health, wellness, and performance [12,13]. Hence, in the current study, percentiles of WB were also used to categorize the population and results showed that the TBW was greater in females with higher WB, indicating that it might also affect body composition. 

It is important to notice that overweight and obesity prevalence obtained in the current study was considerably lower than the available data for the general population of previous studies [2]. In particular, results of the ANIBES study [51] in a population group aged 18-40 years, showed that the prevalence of overweight and obesity was 52.6% and 36.2% in men and women, respectively. These differences could be attributed to the wide age range considered in this study. However, according to the 2017 Spanish National Health Survey [2], the prevalence of overweight and obesity in population aged 18–24 years was 28% and 16.3% in men and women, respectively. These results are similar to those obtained in the current study. 

Nevertheless, as it occurs in the general population, and in the ANIBES study, there were significant differences in the prevalence of overweight and obesity between genders, being higher in males compared to females. Concerning this matter, it is important to consider that the participation in the study was entirely voluntary and the collection of anthropometric measurements may have constituted a barrier in certain population groups, which could be influencing the previous data. 

Results of water consumption obtained in the current study were slightly higher when compared to others, which is mainly due to the methodology used for data collection. In this case, the recently validated questionnaire “HSQ” was used, which was specifically designed to recall water consumption, elimination and thus estimate WB [34,35], whereas most of the previously available surveys used unspecific questionnaires, which leads to an underestimation of WI in most of the cases [28,52,53]. Furthermore, it has been observed that absolute WI was higher in males of this study than in females, although a higher volume of WI from food, higher WB, as well as higher volume of WI from all sources normalized by body weight was reported in the group of females. Thus, although absolute WI was higher in males than in females, when the body size was considered, it was higher in females compared to males. Results of previous studies showed that women tend to exhibit a healthier pattern of eating and food choices than men, whose dietary choices usually are likely to be less healthy [54,55,56]. These results could reflect as well the positive effects of water consumption in weight management.

Strengths of this study included the novelty, the sample size, the use of a specific validated hydration questionnaire, and the quality of the anthropometric data collected. Moreover, it is important to highlight the new and accurate methodology used for food collection. However, the most important limitation refers to the differences in sample size between genders, as well as to the sample homogeneity: According to the International Standard Classification of Education [57], the socioeconomic level of all the participants included in the study is “high”, a fact which is related with lower prevalence of overweight and obesity [58,59]. Likewise, another important limitation is the impossibility of the designed study to differentiate the effect of each type of beverage on body weight and body composition. In addition, underreporting and misreporting of water and other beverage intakes by subjects could have an influence in overall WI and WB. When it comes to dietary patterns, and according to literature, women are more likely to underreport than men, and underreporting is more common among overweight and obese individuals [60]. However, in the present study, underreporting and misreporting was not assessed. Finally, subjects’ socioeconomical status could have an influence as it is related to better BMI across the population [61]. However, we did not assess our data taking these factors into account.

## 5. Conclusions

Our results indicate that fluid intake plays a positive role in body composition and body weight. Therefore, individualized water intake and water balance strategies could be useful in weight management and in the prevention of overweight and obesity in healthy young adults.

## Figures and Tables

**Table 1:** Anthropometric characteristics of participants from the study.

Anthropometric Variables	Males (*n* = 121)	Females (*n* = 237)	*p* Values
Weight (Kg)	73.6 (71.8–75.3)	57.4 (56.4–58.4)	0.000
Height (cm)	177.3 (176.1 - 178.5)	164.4 (163.6–165.2)	0.000
BMI (Kg/m^2^)	23.4 (22.9–24.0)	21. 2 (20.9–21.5)	0.000
WC (cm)	79.1 (77.8–80.3)	67.5 (66.8- 68.2)	0.000
TBW (%)	55.5 (54.7–56.2)	50.2 (49.7–50.7)	0.000
TBW (L)	40.5 (39.8–41.2)	28.6 (28.3–28.9)	0.000
FBM (%)	17.4 (16.7–18.1)	27.4 (26.8–27.9)	0.000
FBM (Kg)	13.0 (12.2–13.8)	15.9 (15.4–16.5)	0.000
LBM (Kg)	60.6 (59.5–61.8)	41.6 (41.0–42.2)	0.000
DLBM (Kg)	19.9 (19.4–20.5)	12.9 (12.6–13.3)	0.000

Results are presented as mean and confidence interval; *p* values derived through Student’s *t* test. (BMI—body mass index, WC—waist circumference, TBW—total body water, FBM—fat body mass, LBM—lean body mass, DLBM—dry lean body mass).

**Table 2:** Water intake from all sources, water elimination, water balance, and water intake from all sources normalized by body weight, obtained by the hydration status questionnaire and sorted by gender.

	Males (*n* = 121)	Females (*n* = 237)	*p* Values
Drinking water (mL/day)	1569.9 (1449.0–1690.9)	1421.7 (1346.2–1497.2)	0.033
Water from beverages (other than water) (mL/day)	2615.9 (2454.1–2777.6)	2297.1 (2196.1–2398.2)	0.001
Water from food (mL/day)	674.7 (607.5–742.0)	780.9 (733.8–827.9)	0.011
Water intake (mL/day)	3290.6 (3111.0–3470.2)	3078.0 (2958.3–3197.8)	0.048
Total water loss (mL/day)	2653.0 (2468.2–2837.8	2181.9 (2097.1–2266.8)	0.000
Water balance (mL/day)	637.6 (443.3–831.9)	896.1 (765.2–1026.9)	0.027
Water intake/weight (mL/Kg)	45.1 (42.6–47,6)	54.4 (52.2–56.6)	0.000
Drinking water/weight (mL/ Kg)	21.5 (19.9–23.2)	25.1 (23.7–26.5)	0.002
Water from beverages/weight (mL/Kg)	35.8 (33.6–37.9)	40.5 (38.7–42.4)	0.002
Water from food/weight (mL/Kg)	9.3 (8.3–10.3)	13.8 (13.0–14.7)	0.000

Results are presented as mean and confidence interval; *p* values derived through Student’s *t* test.

**Table 3:** Correlation between water intake normalized by body weight from all the sources analyzed with anthropometric and body composition variables of males.

Anthropometric Variables	Drinking Water/Weight (mL/Kg)		Water from Beverages/Weight (mL/Kg)		Water from Food/Weight (mL/Kg)		Water Intake/Weight (mL/Kg)	
	*r*	*p* Values	*r*	*p* Values	*r*	*p* Values	*r*	*p* Values
Weight (Kg)	−0.169	0.063	−0.162	0.076	−0.199	0.028	−0.220	0.015
BMI (Kg/m^2^)	−0.084	0.358	−0.094	0.304	−0.173	0.058	−0.150	0.100
FBM (%)	−0.104	0.256	−0.106	0.248	−0.166	0.068	−0.158	0.084
FBM (Kg)	−0.147	0.108	−0.145	0.113	−0.186	0.041	−0.200	0.028
WC (cm)	−0.167	0.069	−0.176	0.055	−0.157	0.087	−0.215	0.018
TBW (%)	0.177	0.053	0.165	0.071	0.210	0.021	0.226	0.013

Results are presented as Pearson’s (*r*) correlation coefficient. (BMI—body mass index, FBM—fat body mass, WC—waist circumference, TBW—total body water).

**Table 4:** Correlation between water intake normalized by body weight from all the sources analyzed with anthropometric and body composition variables of females.

Anthropometric Variables	Drinking Water/Weight (mL/Kg)		Water from Beverages/Weight (mL/Kg)		Water from Food/Weight (mL/Kg)		Water Intake/Weight (mL/Kg)	
	*r*	*p* Values	*r*	*p* Values	*r*	*p* Values	*r*	*p* Values
Weight (Kg)	−0.218	0.001	−0.263	0.000	−0.262	0.000	−0.318	0.000
BMI (Kg/m^2^)	−0.184	0.005	−0.216,	0.001	−0.251	0.000	−0.275	0.000
FBM (%)	−0.138	0.034	−0.229	0.000	−0.202	0.002	−0.267	0.000
FBM (Kg)	−0.192	0.003	−0.267	0.000	−0.258	0.000	−0.320	0.000
WC (cm)	−0.144	0.027	−0.183	0.000	−0.223	0.001	−0.237	0.000
TBW (%)	0.206	0.001	0.290	0.000	0.227	0.000	0.327	0.000

Results are presented as Pearson’s (*r*) correlation coefficient. (BMI—body mass index, FBM—fat body mass, WC—waist circumference, TBW—total body water).

**Table 5:** Differences in water intake variables and water balance according to body mass index and sorted by gender.

	Males			Females		
	Underweight (*n* = 4)	Normal Weight (*n* = 89)	Overweight/Obesity (*n* = 28)	Underweight (*n* = 24)	Normal Weight (*n* = 197)	Overweight/Obesity (*n* = 16)
Drinking water (mL/day)	1091.9 (914.8–1269.2)	1510.5 (1385.8–1635.2)	1827.2 (1491.5–2162.8)	1341.8 (1099.2–1584.5)	1429.2 (1348.2–1510.1)	1448.9 (1027.8–1869.9)
Water from beverages (mL/day)	1797.1 ^a^ (1287.4–2306.8)	2537.1 ^ab^ (2377.8–2696.3)	2983.3 ^b^ (2515.8–3450.8)	2089.7 (1824.1–2355.2)	2310.9 (2200.1–2421.7)	2439.1 (1910.8–2967.4)
Water from food (mL/day)	764.0 (202.5–1730.6)	661.2 (584.2–738.3)	704.8 (559.5–850.2)	724.4 (606.9–841.8)	793.2 (739.5–846.9)	713.8 (553.6–873.99
Water intake (mL/day)	2561.1 (1286.9–3835.2)	3198.3 (3019.8–3376.3)	3688.2 (3176.3–4200.1)	2814.0 (2500.4–3127.6)	3104.1 (2971.3–3236.9)	3152.9 (2584.0–3721.8)
Water balance (mL/day)	−345.0 (–2203.2–1513.2)	696.4 (489.9–902.9)	591.1 (80.1–1102.0)	731.3 (292.9–1169.7)	907.7 (761.6–1053.7)	1000.4 (581.3–1419.6)
Drinking water/ body weight (mL/Kg)	19.1 (15.1–23.1)	21.7 (19.8–23.6)	21.4 (17.4–25.4)	27.9 ^c^ (22.6–33.2)	25.2 ^cd^ (23.7–26.7)	19.1 ^d^ (14.4–24)
Water from beverages/body weight (mL/Kg)	31.6 (20.7–42.6)	36.2 (33.9–38.8)	34.7 (29.4–40.0)	43.3 (37.6–48.9)	40.8 (38.8–42.9)	32.7 (27.1–38.3)
Water from food/body weight (mL/Kg)	13.3 (3.1–29.7)	9.4 (8.3–10.5)	8.3 (6.5–10.0)	15.0 ^e^ (12.5–17.4)	14.0 ^e^ (13.1–15.0)	9.7 ^f^ (7.6–11.8)
Water intake/body weight (mL/Kg)	44.9 (22.3–67.6)	45.8 (43.0–48.6)	43.0 (37.1–48.9)	58.3 ^g^ (51.6–65.0)	54.9 ^g^ (52.4–57.4)	42.4 ^h^ (36.2–48.7)

Data reported as means ± standard error of the mean per group. Different superscript lowercase letters indicate statistical significance in each row (*p* ≤ 0.05) assessed through ANOVA test. Body mass index cut offs: Underweight: < 18.5 kg/m^2^; normal weight: 18.5–24.9 kg/m^2^; overweight/obesity > 25 kg/m^2^.

**Table 6:** Differences in anthropometric and body composition variables according to water intake per body weight percentiles in females.

Percentiles	Percentiles of Water Intake Normalized by Body Weight (mL/Kg)			
	<p25 (*n* = 63)	p25–p50 (*n* = 62)	p50–p75 (*n* = 59)	>p75 (*n* = 53)
Weight (Kg)	60.86 ^a^ (58.4–63.3)	57.8 ^a^ (55.8–59.9)	58.4 ^a^ (56.2–60.5)	54.2 ^b^ (52.7–55.7)
BMI (Kg/m^2^)	22.3 ^c^ (21.5–23.1)	21.3 ^cd^ (20.7–22.0)	21.4 ^c^ (20.9–22.0)	20 ^d^ (19.8–20.9)
FBM (%)	29.1 ^e^ (27.7–30.6)	27.4 ^ef^ (26.2–28.5)	27.9 ^ef^ (26.9–28.8)	26.0 ^f^ (25.1–27.0)
FBM (Kg)	18.0 ^g^ (16.4–19.5)	16.0 ^gh^ (14.9–17.1)	16.5 ^g^ (15.4–17.6)	14.2 ^h^ (13.4–15.1)
WC (Cm)	69.9 ^i^ (67.9–71.9)	67.2 ^ij^ (65.7–68.7)	68.2 ^ij^ (66.6–69.8)	65.8 ^j^ (64.8–66.8)
LBM (Kg)	42.8 ^k^ (41.6–49.7)	41.9 ^kl^ (40.8–43.1)	41.9 ^kl^ (40.7–43.2)	40.2 ^l^ (39.1–41.3)
DLBM (kg)	13.8 ^m^ (13.1–14.4)	13.1 ^mn^ (12.4–13.7)	13.1 ^mn^ (12.5–13.8)	12.2 ^n^ (11.7–12.7)
TBW (%)	48.4 (47.1–49.7)	50.1 ^p^ (49.1–51.2)	49.7 (48.8–50.6)	51.7 ^p^ (50.9–52.2)

Data reported as means ± standard error of the mean per group. Different superscript lowercase letters indicate statistical significance in each row (*p* ≤ 0.05) according to ANOVA test. Percentiles of water intake normalized by body weight: < p25 = 39.6 mL/Kg; p25–p50 = 39.6–48.9 mL/Kg; p50–p75 = 49.0–60.1 mL/Kg; >p75 = 60.2 mL/Kg. (p—percentiles; BMI—body mass index, WC—waist circumference, TBW—total body water, FBM—fat body mass, LBM—lean body mass, DLBM—dry lean body mass).
